# Linking humans, their animals, and the environment *again*: a decolonized and more-than-human approach to “One Health”

**DOI:** 10.1051/parasite/2020055

**Published:** 2020-11-03

**Authors:** Nicolas Lainé, Serge Morand

**Affiliations:** 1 UMR 208 – IRD/MNHN – “Patrimoines Locaux Environnement et Globalisation”, Muséum National d'Histoire Naturelle, Département Homme et Environnement 57 rue Cuvier CP 51 75231 Paris Cedex 05 France; 2 DIM OneHeath Institut de Recherche sur l’Asie du Sud-Est Contemporaine (IRASEC) Bangkok 10330 Thailand; 3 CNRS-ISEM Université de Montpellier CIRAD-ASTRE, Faculty of Veterinary Technology, Kasetsart University Bangkok 10900 Thailand

**Keywords:** One Health, (Multispecies) Ethnography, Knowledge, Decolonization, Global health

## Abstract

This article considers a broad perspective of “One Health” that includes local and animal knowledge. Drawing from various colonial efforts to link human, animal, and environmental health, it first shows that the current “One Health” initiative has its roots in colonial engagement and coincides with a need to secure the health of administrators (controlling that of local populations), while pursing use of resources. In our contemporary period of repeated epidemic outbreaks, we then discuss the need for greater inclusion of social science knowledge for a better understanding of complex socio-ecological systems. We show how considering anthropology and allied sub-disciplines (anthropology of nature, medical anthropology, and human-animal studies) highlights local knowledge on biodiversity as well as the way social scientists investigate diversity in relation to other forms of knowledge. Acknowledging recent approaches, specifically multispecies ethnography, the article then aims to include not only local knowledge but also non-human knowledge for a better prevention of epidemic outbreaks. Finally, the conclusion stresses the need to adopt the same symmetrical approach to scientific and profane knowledge as a way to decolonize One Health, as well as to engage in a more-than-human approach including non-human animals as objects-subjects of research.

## Abstract (fr)

Cet article envisage une perspective élargie de « One Health » (« une seule santé ») qui inclut les connaissances locales et celles des animaux. S’inspirant de divers efforts coloniaux pour relier la santé humaine, animale et environnementale, il montre d’abord que l’initiative « One Health » prend ses racines durant la période coloniale et coïncide avec la nécessité de garantir la santé des administrateurs (contrôlant celle des populations locales) tout en poursuivant l’exploitation des ressources. Dans notre période contemporaine d’épidémies à répétition, il aborde ensuite la nécessité d’une plus grande inclusion des travaux des chercheurs en sciences sociales pour une meilleure compréhension des systèmes socio-écologiques complexes. L’article montre comment la mobilisation de l’anthropologie et des sous-disciplines connexes (anthropologie de la nature, anthropologie médicale et études homme-animal), met en évidence les connaissances locales sur la biodiversité ainsi que la façon dont les chercheurs en sciences sociales l’étudient en relation avec d’autres formes de connaissances. Prenant en compte les approches récentes développées dans le domaine, notamment l’ethnographie multi-espèces, il vise alors à inclure non seulement les connaissances profanes mais aussi les connaissances non-humaines pour une meilleure prévention des épidémies. La conclusion souligne la nécessité de mettre sur une même ligne symétrique les connaissances scientifiques et profanes comme moyen de décoloniser One Health, ainsi que de s’engager dans une approche désanthropocentrée en incluant les animaux non humains comme objet-sujet de recherche.

## Introduction: The colonial root of “One Health”

The “One Health” initiative, a tripartite collaboration launched in 2008 between the World Health Organization (WHO), the World Organisation for Animal Health (OIE), and the Food and Agriculture Organization of the United Nations (FAO), advocates a rapprochement between human and veterinary medicine for a better understanding of infectious diseases that spread across species and how they interact in the environment. While tracing the history of this convergence, scholars often acknowledge American veterinary epidemiologist Calvin W. Schwabe [42] for his proposal of “One Medicine” [58]. In fact, what is currently referred to as the One Health initiative traces its roots back even further, to the colonial era. This period is rich in attempts to link human, animal and environmental health. Importantly, this was also a period marked by a strong distinction between the colonial science administrators and the local populations they controlled.

In this paper, we first intend to further explore the colonial origin of One Health and show how the current holistic approach as initially promoted resonates in our contemporary period. Let us clarify that we are not veterinarians nor medical doctors ourselves, but an anthropologist interested in human-animal relations and a health ecologist sharing a common interest in studying the links between health, societies, and biodiversity. Drawing on recent developments in social science methodologies, we then aim to look into how the entanglement of all living beings in a socio-ecological system could be better taken in consideration for future research in this area.

Back in 1959, a striking quote from Thomas Logan, a doctor of the Californian “New Frontier”, highlights the fact that complex links between the environment and health were recognized in public health early on: “*A knowledge of the etiology of diseases can best be attained by studying the affections of different localities in connection with every condition and circumstance calculated to operate prejudicially or otherwise upon the health of the inhabitants. Such philosophical investigation is particularly useful in tracing the modifications diseases may undergo from the agency of causes of a local or special character; and being also calculated to elucidate the relationship of diseases to climate, to the prevailing geological formations — the fauna, the vegetables, the minerals, the waters, which vary with the earth’s crust,…*” (Thomas Logan, Transactions of the American Medical Association, 1859, quoted in Nash [35]). At that time, Thomas Logan and his colleagues were confronted with diseases affecting their fellow European citizens who were colonizing the “New Frontier” habitats – that is to say without including native Amerindian populations. To tackle diseases, Logan proposed an environmental and geographical approach to human health. He was certainly aware of and inspired by the writings of Alexander Von Humboldt, the founder of modern biogeography with his “*Essay on the Geography of Plants*” [55].

As mentioned by Tilley [50], the era of “interventionist” colonialism encompassed agriculture, public health, natural resource use, disease control, labor recruitment, and conservation measures, all developing in the first half of the twentieth century.

As for forest exploitation, colonial administrations introduced scientifically based policies for the management of their Empires. As the Indian historian Ravi Rajan points out, the establishment of forest departments, and other agencies, “*resulted in the creation of a homogeneous and assertive pancolonial community of foresters*” [38]. In fact, as demonstrated by the environmental historian Richard Grove, colonial forestry has to be seen as the root and origin of environmentalism [15]. At that time, all European foresters shared a common representation that new forestry should preserve forests from mis-management by local populations, who were blamed for forest degradation, a discourse that has continued until recently, taking the form of neocolonialist conservation. For example, French foresters saw themselves as “engineers” concerned with the impacts of deforestation on watersheds by putting forward the connection between “forest cover, healthy watersheds, and agricultural productivity” [38], an interesting link echoing the more recent “healthy landscapes” [1].

Looking at the socio-economic development of colonies, Julian Huxley is probably one of the most preeminent British activists of new colonial science [50]. In the 1920’s, he made a trip to Africa sponsored by the British Committee on Native Education in Tropical Africa. The committee asked him how biological science and knowledge of the natural world might be integrated into general educational efforts. Huxley replied to the Committee by suggesting that African study centers should adopt an ecological framework: “*At the present moment, it is clear that many if not most problems of applied biology can only be satisfactorily solved by reference to a background of ecological ideas, by whose aid the interrelations of different branches of biological science can be studied*” (quoted by Tilley [50]). Huxley also claimed that “*it is often possible for the ecologist to point to this or specialist new lines of approach to his particular problem – disease of man or of domestic animals – may prove to be correlated with a cycle of abundance and scarcity in some wild animals . . . game migrations or … climatic cycles or variations in mineral content of foodplants.*” (quoted by Tilley [50]). On that, Huxley emphasized the importance on a “*close liaison between the Department of Ecology and any Anthropological work prosecuted in the School of African Studies, and with medical work bearing on Africa*.” A proposal resumed by Tilley as: “*This triumvirate—ecology, anthropology, and medicine— was central to colonial Africa’s economic and social development*”.

**Figure 1:**
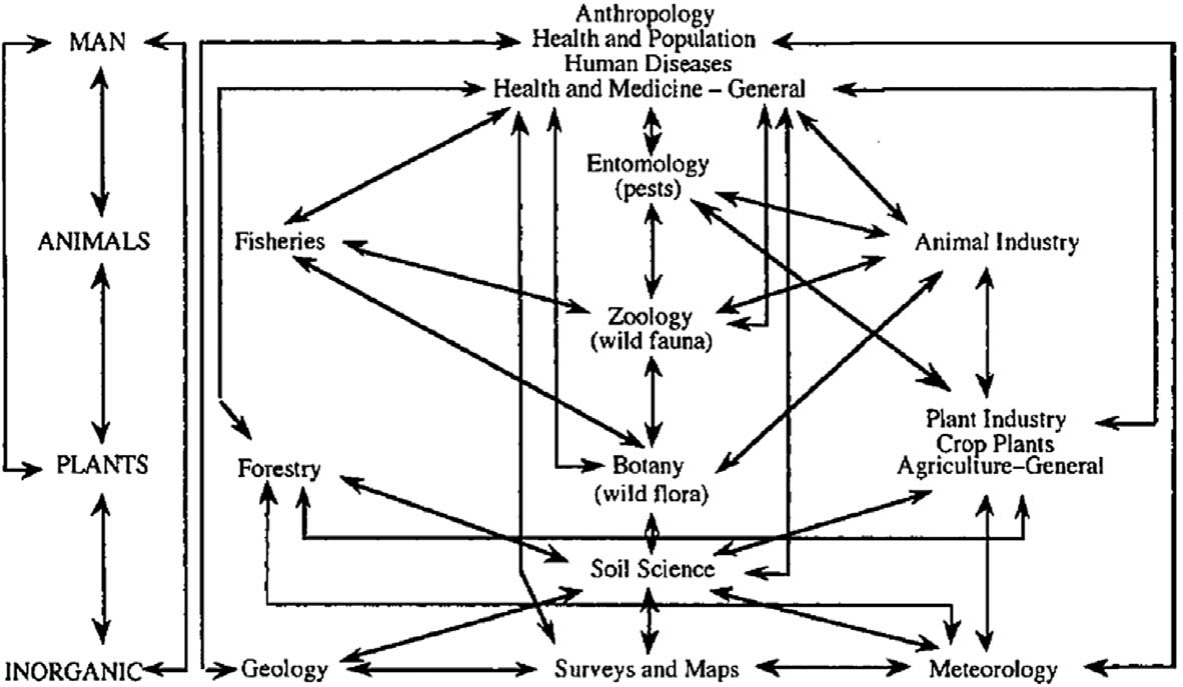
The colonial scientific network of environmental management in Worthington [56] (see also Tilley [50] , Morand & Lajaunie [33]).

In the 1930s, The African Research Survey emerged as a network of academics and officials, i.e., the London and Liverpool Schools of Tropical Medicine, the Imperial Forestry Institute in Oxford, and the Imperial Agricultural Bureaux). Under its director Malcolm Hailey and the scientific adviser Edgar Barton Worthington, the survey authored “*Science in Africa*”, a book summarizing the works carried out [56]. One diagram included is fascinating (Fig. 1); even more is the way it was presented by Worthington [56]: “*The picture really presented by Africa is one of movement, all branches of physical, biological and human activity reacting on each other, to produce what biologists would refer to as an ecological complex*” (quoted by Tilley [50]). For contemporary health policy makers this type of figure is very striking and resembles many of today’s.

Environmental influences on health were analyzed by two French geographers, Maximilien Sorre [48], who was credited with the concept of the pathogenic complex, and Jacques May [31], the founder of modern medical geography. Sorre [48] argued that the emergence of diseases depends on physical, biological and social factors and more specifically on the climate, the natural biological environment and the anthropo-geographical environment (see Oppong & Harold [36]). For Sorre, the environmental conditions, the living conditions of the pathogen and the characteristics of individuals influence the appearance of a disease. May, who started his career as a medical doctor in French Indochina, focused on the role of the environment in the formation of human diseases and the importance of geography in mapping pathological trends. May provided a theoretical framework for studying the environment and geographical factors (in his words, “geogenetics”) of pathogen emergence. He continued his career in the United States where he worked for the United States Agency for International Development (USAID) and the WHO.

At the end of World War II, and the start of the decolonization period, Julien Huxley, after co-founding the World Wide Fund for Nature (WWF) and initiating the creation of the International Union for Conservation of Nature (IUCN), was appointed as the first director of the newly created United Nations Educational, Scientific and Cultural Organization (UNESCO). Later on, in 1968, the links between societies, health and biological conservation were addressed at the UNESCO Biosphere Conference, in which the scientific basis for the rational use and conservation of biosphere resources were drafted. In terms of health, the loss of biological diversity was directly associated with the deterioration of physical and mental health: “*Whether the challenges come from physical or social forces, the diversity of environments is of crucial importance for the evolution of man and his societies because the ultimate results of a stereotyped and equalized environment can be and often are an impoverishment of life, a progressive loss of the qualities that we identify with humanness and a weakening of physical and mental health. Our policy should be to preserve or to create as many diversified environments as possible*” [52]. Interestingly, in Recommendation 3 “Research on Human Ecology” of the final report of the conference after considering that “*man is an integrated part of most ecosystems, not only influencing but being influenced; that his physical and mental health, now and in the future, are intimately linked with the dynamic systems of natural objects, forces and processes that interact with the biosphere and including also the man’s culture*”, made the recommendation “*that continuing and intensified research should be undertaken on the ecology of human diseases, with special references to those associated with environmental change and to the zoonotic diseases arising from the interactions between man and the animal*”. What is important to emphasize is that this recommendation called for the implementation of an ecology of zoonotic diseases that should integrate the problematic of environmental changes and consider human culture.

For contemporary researchers engaged in the understanding of various social, ecological, and biological factors related to the emergence of diseases, the writings of Logan, Huxley, and Worthington in the final report of the UNESCO conference of 1968 appear to be very modern. So much so, that they could have been written today by any of the current international organizations involved in the One Health initiative [2]. However, one can ask why these writings of the late 19th century or the middle of the 20th century that appear so relevant have disappeared from our contemporary scientific writings (see [9])? Scientific researchers involved in “One Health” should integrate in their discourse the fact that a large part of the rhetoric they use is not new but deeply rooted in the colonial sciences that aimed at developing local societies, their health, and the health of their livestock, as well as their economies by favoring their integration into the Empire market as that time, and to the global market today.

Interestingly, the network presented above, which does not exclude any of our “modern” scientific disciplines, puts anthropology at the top of the chart. This is something hardly taken into consideration when assessing policy of preparedness or response to zoonotic outbreak that regularly flourish on a global scale nowadays, with the fear of a new pandemic (see Box 1). Anthropology, a discipline that found its origin in the colonial period itself, seems to have been banned for a long time from public health engagement, something that may be explained by its initial racial theories serving colonial wills. Often considered as the “daughter of colonialism”, anthropology has been considered to serve the colonial administration. To move away from this view, in France, the use of the terms “compared sociology” or “ethnology” replaced the term “anthropology” in academia for decades in the 20th century. It was then been reiterated by Claude Lévi-Strauss after the Second World War which introduced the term “Social-Anthropology” in the country.

Box 1 **Ongoing global crises**

**Figure 2:**
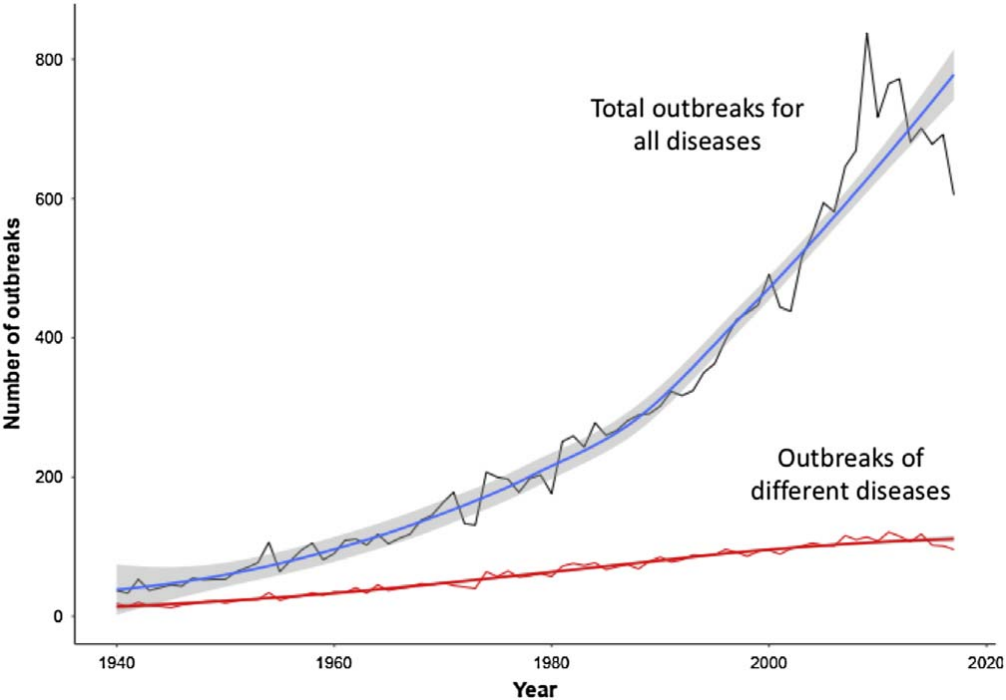
Number of infectious diseases presenting outbreaks globally over the last 60 years from GIDEON (Global Infectious Diseases and Epidemiology Network, www.gideononline.com).

Since the end of the 20th century, we have observed an increase in the number of emerging infectious diseases [19, 45] mainly related to climate change, land use change, growth of global trade, and biodiversity loss. Biodiversity loss through altered landscapes due to urbanization and agricultural intensification appears to be linked to higher disease risks, with the emergence of novel pathogens resulting from increased interactions between wildlife, domesticated animals, and humans [16, 18, 28]. Such infectious diseases have led to an increasing number of global outbreaks with a slight but constant appearance of new pathogens worldwide. Another trend observed is the homogenization of global parasite distribution, which began around 1960 [46]. Using network analysis, a striking decrease of the modularity of the country-pathogen networks has also been observed [37], suggesting that outbreaks of infectious diseases are increasingly shared among an increasing number of countries. That is to say that today, an outbreak of a given infectious disease has a greater chance of spreading among a larger number of countries due to globalization. These above patterns strongly suggest that global changes are affecting the global epidemiological environment, mostly by favoring the spread of infectious diseases among countries and by increasing the risks of pandemics [49]. An echo today with the emergence of the 2019-nCoV originating in China, and rapidly spreading from this country to a global scale (Fig. 2).

## Results and discussion

### Biological and cultural diversity: a (still) missing link for better health

In the context of repeated global health crises, what does it mean today to encompass into a single approach human, animal, and environmental health as highlighted by colonial administrators and currently promoted by “One Health” initiatives? While acknowledging the past colonial view, it is important to remove it from current thinking and relations with populations. Instead, the aim should be to engage with new forms of exchange based on dialogue and mutual collaboration rather than domination. This way, apprehending health as “one” primarily requires us to renew the appreciation of knowledge possessed and implemented by local populations of their immediate environments. The latter have much to say about the current state of knowledge on biodiversity as well as the way to manage it.

Thanks to their local knowledge, their approach and management of territories and resources, local populations are essential actors in meeting the challenges related to global health and environmental risks. This is particularly true as it meets the current requirements of research ethics. An extension of the 1992 International Convention on Biological Diversity signed in Rio de Janeiro, the Nagoya Protocol has governed access to genetic resources (animal, human, and microbial genomes) since taking effect in 2014. This protocol emphasizes the need to involve local populations in research so that they have access to scientific knowledge, participate in building such knowledge, and share in its benefits.

Again, back in 2008, a World Bank report insisted on the fact that indigenous territories encompass up to 22% of the world’s land surface, and hold about 80% of global biodiversity [47]. The role of local knowledge in managing and maintaining a high level of biodiversity was already acknowledged years ago on the occasion of Convention on Biological Diversity (CBD) in 1992 in Rio de Janeiro. In particular, Article 8J of this convention emphasized the preservation of local knowledge and know-how for the conservation and sustainable management of biodiversity. It states to respect, preserve and maintain knowledge, innovations and practices of indigenous and local communities embodying traditional lifestyles relevant for the conservation and sustainable use of biological diversity.

We should add that the rich immediate and surrounding environment that the local population are living in provides not only for their basic daily needs, but also immediate resources for medicines, and inspiration for their cultural and spiritual activities. Biodiversity is an integral part of these societies, reflecting a particular way of living and representing the world, known as cosmology. Examples include the Kasua in Papua Guinea [6] who have borrowed some of their attitudes – expressive, sexual, technical, ceremonial, even ritual – from animals co-evolving in their shared forest, or the Aїt Ba’amran communities in South Morocco, and the Quechua populations of Peruvian Amazonia [44] who employed natural elements to transmit their culture. For these peoples, there is no boundary between nature and culture as projected in western dualistic ontology. For them, biodiversity is intrinsically linked with their own culture and identity. But we must be precise before continuing, our will to highlight to role of local knowledge does not involve naively idealizing the knowledge and know-how possessed by communities but considering the way they are sometimes forgotten or smothered by global measures, they could rather be sentinels for better anticipation and management of health and environmental crises, as notably shown by Ruhlmann [40] with Mongolian herders.

Anthropologists (and social scientists in general) are well positioned to respond to this need for in-depth and immersive research. As a people-centered discipline, historically, anthropology accounts for the diversity and complexity of relationships that communities share with their environment. Back in 1962, this knowledge refers to what Claude Lévi-Strauss called the “*science of concrete*” in his book *The Savage Mind* [24]. In the first chapter of the volume, he attempted to characterize two modes of thought, or methods toward acquiring knowledge: the “science of the concrete” or mythical thought, and modern scientific inquiry. His demonstration stressed that both scientific and mythical thought should be understood as valid and that one does not supersede the other. They actually consist of two autonomous ways of thinking, rather than two stages of evolution as thought back in the colonial period. Despite a growing demand for social scientists to tackle global problems nowadays [43], we tend to see calls for contributions from social sciences only *after* an epidemic outbreak in emergency situations. Since the Ebola outbreaks, social scientists have been asked to facilitate the implementation of measures of control and policy, to help in describing the local context, and to help in understanding local risk practices (see Box 2).

Box 2 **Social Scientists and Ebola outbreaks**

In recent years, anthropologists have become valuable stakeholders to address the social, economic, political, and cultural intertwining in epidemic outbreaks. By involving social science researchers who were present or directly engaged in the field, the recent (and many) episodes of Ebola crises across West Africa highlighted the nature of their contributions. Although recent, feedback has demonstrated the value of involving these researchers who used to work closely with affected populations. A recent special issue of the journal “*Anthropology in Action*” (2017) draw up a first – but not exhaustive – inventory of interventions, in particular from researchers in the field of medical anthropology and development anthropology who turned their research into applied anthropology [53]. For the involved researchers this kind of situation also challenged methods of conducting fieldwork. One of the main areas of activity of social scientists in emergency situations is their presence for the promotion of health measures by NGOs or health experts, making such measures understandable and acceptable to local communities. Online networks and platforms dedicated to the multiple dimensions of outbreaks, especially in helping to implement accurate local interventions, have been launched (see http://www.ebola-anthropology.net/ and https://shsebola.hypotheses.org/).

It is clear that one of the roles of social scientists is to mediate between the various knowledges to enable a dialogue between scientists, decision-makers, and local populations, particularly during emergency situations such as epidemic outbreaks where difficult measures are decided. Importantly, while health issues have invaded the public space, particularly those related to the origin of animal diseases, they crystallize opinions and actors involved. Various situations have lead to a misunderstanding of the measures (slaughter) or issues (particularly economic) associated with the management or prevention of health crises. In the livestock sector, for example, there is a widespread reciprocal fear mentality in France between local farmers who fear that wildlife will affect livestock on the one hand, and scientists or conservationists who fear that livestock will affect wildlife, on the other [12]. But should the social scientists be restricted to a role of health promoter, *cultural broker,* or risk communicator? Pursuing with the case of Ebola, a group of scholars [41] directly involved in field during outbreaks insist on their role in the post-crisis period, specifically for the follow-up of patients who experienced the social effects of the disease. For Ebola survivors, this includes understanding the physical, social and psychosocial effects of it. Inputs provide precious feedback on the way patients experienced measures during an epidemic. Such information could certainly be crucial for better adaptation of measures and coordination between global and local health agencies.

Shall we go even further in this step and include *upstream* the inputs of social sciences in the prevention of risks related to animal, human and environmental health? As for anthropologists, this question is closely related to the way they conduct their research, the type of data they collect, and more crucially the approach they employ to collect them.

### How to access local knowledge

Anthropology (or any related discipline) through its approach (field survey over time, bottom-up approach) and its methodological tools (participating and repeated observation of practices, attention to detail, data collection in vernacular language, interviews or life stories) can tell much about the various perspectives on phenomena such as the transmission of diseases from humans to animals, and from domestic to wild animals. Instead of taking the global guidelines that guide local actions as a starting point, most social scientists have in common to engage in a bottom-up approach, using ethnography as the sole method.

This is the case in the anthropology of nature promoted by Descola [8] who challenged the western dualistic view of nature and culture. Changing such perspectives can help in understanding the social and cultural factors that allow pathogens to cross the interspecies barrier locally [14]. As the scientific names of viruses and pathogens are rarely translated into local languages and also hardly make sense for local communities (cf. *undo*), anthropology of nature primarily invites us to shift the focus from the pathogen itself to the construction of the human-nonhuman frontier. Investigations could then focus on how the interspecies frontier is thought to probe the extent to which it does or does not allow the passage and spread of pathogens. Following the ontological perspective of Descola [8], pathogens are then investigated through the interiority and exteriority of beings. For example: is the pathogen present (visible) inside or outside the body of animals? According to those who are affected and exposed to it, through what type of contact could transmission have taken place?

Complementarily to anthropology of nature, medical anthropology helps in appreciating the diversity of points of view on biological phenomena such as the transmission of diseases between humans and animals, and between domestic and wild animals. It is also a well-positioned perspective to engage into multidisciplinary dialogue. As indicated by Panter-Brick and Eggerman [23], medical anthropology “*sits at the intersection of the humanities, social sciences, and biological sciences, seeking to transform our understanding of “what matters” for people in terms of health, well-being, and the environment. Embracing far ranging interests, it generates in-depth knowledge about the ways people understand these issues and frame health-related decisions”.* Research in medical anthropology sheds light on the understanding between the biomedical representation of viruses and/or diseases and their local interpretations. It reveals various conceptions of diseases, different values, and perceptions orienting animal management.

In addition, medical anthropology invites to keep a close eye on policies and relations shared between all actors involved in the field and their links. These links, as we know, are not neutral and include issues of knowledge and ultimately power. Something applies to any situation where different conceptions of health and disease are at stake. For example, while studying elephant tuberculosis surveillance in Laos, Lainé [26] revealed several levels of incomprehension and a lack of dialogue between the local mahouts and veterinarians. There, instead of facilitating exchanges, it has only exacerbated tensions, probably already present, between the various actors involved (NGO, veterinarian, mahout and elephant owner). Lastly, this type of biosecurity device [11] has merely offered new legitimacy to veterinary science over local knowledge.

Considering the importance of domestic animals on our planet [32], the latent risk of epidemic outbreaks, related to our growing dependency on livestock for food, makes human-animal studies [7] and ethnozoology [17] flourishing areas for medical anthropology [4]. Local ethnographies on human-animal relations make it possible to investigate how farmers and people engage daily with animals, as well as their relationships with them in terms of distance and proximity depending on their health situation. For example, during fieldwork, researchers ask how people perceive a risk associated with animal diseases. How do they prevent these risks? Under what conditions are animals considered healthy, in their opinion? Are they under the influence of a good or bad spirit?

Drawing from ethnobiological methods, local ethnography seeks local interpretations of animal diseases, and perceptions of associated zoonotic risks. At the same time, researchers collect local inventories of animal diseases and their treatment using ethnoveterinary and ethnobiological tools. In that direction, advocating for better integration of ethnobiological research – including its subfields such as ethnoveterinary studies and ethnomedicine – into the “*One Health*” agenda, Marsha and Robert Quinian [22] recall how a “One Health” perspective is actually a central part of ethnobiology. Reciprocally, they add that “*One Health would benefit from ethnobiology for its natural and social perspective, consideration of deep connections between indigenous people and their landscapes, and its norm of rapport establishment*” (Quinian [22]).

The recent development of ethnographic methods could even further improve our understanding of complex socio-ecological systems. As we will show below, embracing a multispecies approach to One Health enlarges the scope of the research by including non-humans as a subject/object.

### A multispecies approach to One Health

While we have seen that anthropology could historically be defined as a people-centered discipline, in recent years, an “ontological turn” has offered an enlarged vision for understanding the complex entanglements of humans and animals. Within the ontological turn, research has shown that, far from being automatons or machines, animals act and think in their environment, and they have representational abilities in it. This perspective offers new methodological approaches such as multispecies ethnography [20]. It refers to an approach that aims at considering the agency of nonhumans and their multiple (social, historical, and ecological) connectivities with humans, while challenging the anthropocentric vision upon which ethnography historically depends. Thus, within a “desanthropocentric” perspective, animals are no longer thought of as cut off from the world of humans, but as an integral part of this world, and as actors capable of acting and interacting.

As a more‐than‐human approach, multispecies ethnography is open to perspectives from the natural as well as the social sciences. Applying this perspective to a One Health approach allows us to engage in innovative results which could benefit humans, animals, and their shared environment. Research conducted in this more-than-human approach no longer considers animals as passive objects [25]. Rather, they are themselves actors in shaping and producing knowledge along with humans. This paradigm, adapted to a transdisciplinary history of One Health, has recently revealed how animals have shaped medical knowledge [57].

More crucially, what is interesting in adopting a desanthropocentric perspective is the fact that while conducting field studies, it invites us not to choose between human or animal, but to carefully look at the network of relations they built in their shared environment. From an epistemological point of view, this reversal implies that the primacy of knowledge should no longer be granted only to humans. It then gives a prominent place to interspecific interaction and dynamics, thought reciprocally. Finally, conducting a multispecies ethnography of human-animal relations allows the researcher to go even further by discussing the notion of local knowledge and investigating how it could be applied to animals themselves.

Relying on local knowledge, it is possible to explore animal exploitation of resources following what anthropologist Florence Brunois [5] has called ethno-ethology. She encourages researchers to conduct an “*ethnography of how individuals perceive and conceive, in the course of their interaction with them, the behaviour of living beings and how they react to these behaviours*” (Brunois [5], p. 34). In the field, this means accessing animal knowledge and understanding of their immediate environment through the mediation of the people in charge of these animals, in particular how they perceive the said behaviour. This includes, for example, asking them about their knowledge of the plants consumed by animals, or following them through the forest or grazing areas to directly observe the plants or any other plant resources (root, branch, fruit, leaf, vine, bark) consumed by the animals.

Conducting these types of studies requires a strong multidisciplinary approach. In this direction, Krief and Brunois-Pasina [21], primatologist and anthropologist, respectively combined their approach to understand the co-evolution of great apes and humans in the Kibale region of Uganda. Their results show that animals are co-producers of shared medical knowledge with humans.

Also in this way, investigating ethnoveterinary practices on pachyderms in Laos, Lainé [27] showed that according to mahouts, elephants have a rich knowledge of the forest world, which they express by looking for specific plant specimens for food and healing. In the Tai-Lue villages in the northwest of the country, the health and care of these animals is based on local ethnoveterinary practices using local plants, to which must be added an essential element: respect for the knowledge of the elephants themselves, who are capable of self-medication. That is, if people provide them with the plants they need for a healthy diet, they are aware that elephants are able to supplement them if necessary thanks to the abundant diversity of the spaces they cross in their company. Such aspects of elephant health management in villages are considered an integral part of the system of care for these animals. There, mahouts do not pretend to control every aspect of elephant diet and care. According to them, the forest is the equivalent of a pharmacy for the elephants; they find many medicines there. Adding that when they are sick, elephants would prefer to stay alone, without seeing any humans, either their owner or the veterinarian, and that finally the forest is the place where the animal was *sabai* (“healthy”). For example, it is possible to observe an elephant that is tired or thinner, especially after several days of work in the forest. Their morphology can also vary. Everyone agrees that once the task is accomplished, when they leave their elephants at rest, free to roam in the forest, it only takes a few days for them to regain their healthy weight and shape.

The ethnographic survey on human-elephant daily life highlights the interdependency of elephants with local populations they work with, particularly in terms of health and wellbeing. The results of this research first show a concordance in the ritual treatment of humans and elephants (protection by the same household spirit, collective ceremony). Secondly, the collection of information on the diet of elephants highlights a possible convergence of plant use between human and animal health.

This last example not only allows a decentralization of viewpoints on the world from human to animal, but also engages research in a more dynamic and inter-relational perspective, while recalling the necessary holistic approach for research on health and infectious diseases. By giving a primary voice to local knowledge, these examples illustrate how current anthropological research, by focussing on human-animal relations, reverses the order of relations established by colonial veterinarians or doctors, keeping a holistic approach of health. Here, local populations are no longer under the domination of administrators, but appear to be crucial mediators between various actors, including animals themselves.

As we have seen, today’s global economic changes have created a higher demand for livestock and meat production worldwide, which has resulted in homogenization and intensification of human-animal interactions. To counter square such trends, and to limit the loss of biological diversity, there is a need to instigate dialogue between ethnosciences, biodiversity, and health by promoting cultural biodiversity [29]. At the same time, innovative solutions should be purposed, in particular by seeking to reconstruct local veterinary pharmacopoeias based on veterinary scientific knowledge, local pharmacopoeias, and also by adding a third component: the knowledge possessed by animals of their environment and that are capable of self-medication. Operating this epistemological change in how science is “done” leads us to rethink the way research is conducted, including its ethical considerations (see Box 3).

Box 3 **Rethinking ethics**

Alongside the “One Health” approach, in recent years, several discourses on ethics have been produced. The rise of ethical responses to public health crises have been referred to as “One Bioethics” [51], “One Health ethics”, and “Global Health ethics” [13]. Yet, to date, there is no consensus among bioethicists on what this means [54]. As emphasized by Morand and Lajaunie [34], ethical reflection in the field of biodiversity and health requires us to examine the relevant scientific domains (i.e., biology, ecology, evolution, human medicine, animal medicine, anthropology, and juridical science), their epistemology, and the need for scientific pluralism. The latter being essential to establish genuine interdisciplinarity and requires the values, practices, and impact of each constituent field to be evaluated.

Thus, well-established “Global Health ethics” is more essential than to build “One Bioethics”, as proposed by the “One Health” approach, or “Planetary health ethics”. As Verweij and Bovenkerk [53] pointed out, “One Bioethics” or “Planetary health ethics” refer more to the domain of meta-ethics which corresponds to a moral belief in “health” and “Planetary health”. The crucial point is the scientific posture adopted in the face of health crises originating from ecological crises, and its implications for studying nature (broadly conceptualized as ranging from a simple mechanism that can be easily fixed to a complex adaptive system that requires care). The recognition that crises are systemic must lead to the development of systemic actions for better earth stewardship and better common health and well-being.

## Conclusion: Towards ecological health solidarity

In today’s globalized epidemiological environment [32] characterized by the emergence and re-emergence of diseases circulating between humans and animals and the rapid decline of biodiversity, social sciences research shows that there is no single “one size fits all” solution to the health and environmental risks that threaten our planet. Case-based contextual studies conducted in close collaboration with local populations are needed to incorporate their understanding of the environment that they know so well. Working in direct collaboration with local communities also means challenging the question of knowledge and the dominant relations behind it. Recently, a global movement of decolonization of knowledge affecting both ecological [10] and health-related issues [39] has emerged. For the whole scientific community, this means engaging in dialogue and taking into account the plurality of points of view and different types of knowledge including their own logics and epistemologies.

More importantly, in the society of risk we are living in, where scientific knowledge is a source of uncertainty [3], humans should no longer be considered as the sole repositories of knowledge imposed on nature. On the contrary, they must learn to collaborate with non-humans. This means changing our view of wildlife and domesticated animals, and not necessary consider them as passive objects, or in the case of health as victims of pathogens or guilty of transmitting them. Potentially, they are co-producers of knowledge on biodiversity. Recent developments in social science methodologies allow us to take the agency of animals and highlight the interdependencies of living beings in shared territories. This type of perspective sheds light on how social and ecological processes interact with each other and build precious ecological solidarity [30] (including plants, animals, microbes, insects and other species) that can help prevent the next epidemic.

## Conflict of interests

The authors declare that they have no conflict of interest.
